# Establishing spatial and temporal patterns in *Microcystis* sediment seed stock viability and their relationship to subsequent bloom development in Western Lake Erie

**DOI:** 10.1371/journal.pone.0206821

**Published:** 2018-11-21

**Authors:** Christine M. Kitchens, Thomas H. Johengen, Timothy W. Davis

**Affiliations:** 1 Cooperative Institute for Great Lakes Research (CIGLR), Ann Arbor, MI, United States of America; 2 National Oceanic and Atmospheric Administration, Great Lakes Environmental Research Laboratory, Ann Arbor, MI, United States of America; 3 Department of Biological Sciences, Bowling Green State University, Bowling Green, OH, United States of America; INRA, FRANCE

## Abstract

This study assessed the distribution, abundance, and viability of pre- and post-overwintering *Microcystis* sediment seed stocks in Western Lake Erie and how these variables are potentially related to past and subsequent bloom formation. We conducted a two-year spatiotemporal survey of vegetative seed stocks in Western Lake Erie, the region where annual algal blooms generally develop. Sediment was collected from 16 sites covering an area of 375 km^2^ and water column depths ranging from 3–9 meters. Sample collection occurred in November 2014, April 2015, November 2015, and April 2016. The abundance of total and potentially-toxic *Microcystis* cell equivalents were determined using quantitative polymerase chain reaction. A series of laboratory experiments using lake sediment were conducted to assess the viability of *Microcystis* vegetative seed stocks. Across all sampling periods, the abundance of total *Microcystis* in the sediment ranged from 6.6 x 10^4^ to 1.7 x 10^9^ cell equivalents g^-1^, and potentially-toxic *Microcystis* ranged from 1.4 x 10^3^ to 4.7 x 10^6^ cell equivalents g^-1^. The percent potentially-toxic *Microcystis* in the sediment ranged from <1% to 68% across all samples. Total *Microcystis* abundance diminished significantly over winter with densities in spring nearly 10 times less than the previous fall. However, despite cell loss from fall to spring, lab experiments demonstrated that remaining non-toxic and potentially-toxic cells were viable after the overwintering period. Further, lab grow-out experiments indicate that potentially-toxic strains recruited at a slightly higher rate than non-toxic strains, and may in part, contribute to the pattern of higher relative toxicity during early stages of the blooms. The abundance and distribution of overwintering cells did not correlate strongly to areas in the lake where subsequent summer blooms were most persistent. However, numerical analysis suggests that recruitment of benthic overwintering populations could help explain a portion of the initial rapid increase in bloom biomass and the spatial extent of this bloom initiation, particularly when recruitment is paired with subsequent growth in appropriate water column conditions.

## Introduction

Approximately 12 million people live in the Lake Erie watershed, with the lake serving as a source of drinking water to 11 million residents [1]. Due to this high population density, intensive agricultural land-use activity, and shallow morphology, Western Lake Erie is plagued by a long history of cyanobacterial harmful algal blooms (HABs) that are detrimental to human health and aesthetic values. In addition, it has been suggested that the deposition of HABs and subsequent bacterial decomposition is contributing to the ongoing hypoxia problem in the central basin in Lake Erie [2–4]. The intensity and spatial extent of HABs in western Lake Erie has increased over the past decade; the years 2011, 2015, and 2017 saw some of the largest HABs on record [5, 6]. In 2014, HAB infiltration into the Toledo water intake resulted in a nearly 3-day ‘do not drink’ advisory for roughly 500,000 residents [7].

*Microcystis* is a genus of cyanobacteria within the Order Chroococcales, of which blooms reoccur annually within the open waters of Western Lake Erie. In the environment, *Microcystis* cells form colonies held together by a mucilaginous matrix [5, 8–14]. Some strains of *Microcystis* also produce hepatotoxins known as microcystins [10, 15–18]. A key feature of *Microcystis* is the presence of gas vesicles, which allows for buoyancy control and reentry into the water column from the sediment [19]. The typical annual cycle of *Microcystis* in temperate regions includes overwintering in the upper layers of sediment, reintroduction into the water column in the spring, summer bloom formation, and autumn settling into the sediments [20].

Previous studies of other temperate lakes indicate that overwintering populations of *Microcystis* possess high survivability and can seed seasonal *Microcystis* blooms [21–26]. However, exactly how much sediment populations contribute to blooms is less clear, especially when considered against fluvial sources. For example, Conroy et al. found that *Microcystis* comprised a large percentage of algal assemblages in the Maumee and Sandusky Rivers in March 2009, suggesting the rivers as a potential source of inocula for summer blooms [27]. However, other studies have demonstrated genetic differences between river and lake forms and strains of microcystin producers, suggesting that river populations are not the ones seeding summer blooms in the lake [16, 25, 28]. Similarly, upstream sources from the Grangent reservoir were shown to have different genotypes than populations sampled downstream in the connecting Loire River [29]. While evidence indicates that both fluvial and benthic sources of *Microcystis* potentially serve as inocula for blooms, existing evidence is unable to definitively show which of the sources is the more significant contributor to bloom events.

Researchers have previously quantified benthic *Microcystis* populations in Western Lake Erie in attempts to understand how benthic populations contribute to bloom development [25, 30]. However, those studies conducted limited spatial and temporal sampling and did not adequately address the potential for spatial gradients in *Microcystis* population. Further, past studies only sampled during a single summer season and did not assess the potential contribution of overwintering benthic populations of *Microcystis* to seeding subsequent summer blooms. To more adequately evaluate the potential for sediments to serve as a source of inoculum, we conducted our study over multiple seasons and for 16 different sampling sites covering an area of approximately 375 km^2^. In addition, laboratory grow-out experiments were conducted to directly assess the viability and growth potential of the fall and over-wintering *Microcystis* cells. Alongside serving as potential source of bloom inocula, lake sediments also play a role in the biodegradation and adsorption of toxins as well as serve as a potential source of nutrients [31–34]. Therefore, abundance and distribution of vegetative *Microcystis* cells were compared to other sediment proxies (e.g. chlorophyll α, phycocyanin, etc) and water column characteristics to evaluate whether there were clear factors driving observed patterns of abundance across space and time. To our knowledge, this is the first study to assess both the abundance and viability of post-overwintering sediment *Microcystis* in Western Lake Erie.

## Materials and methods

### Site description

**Fig 1:**
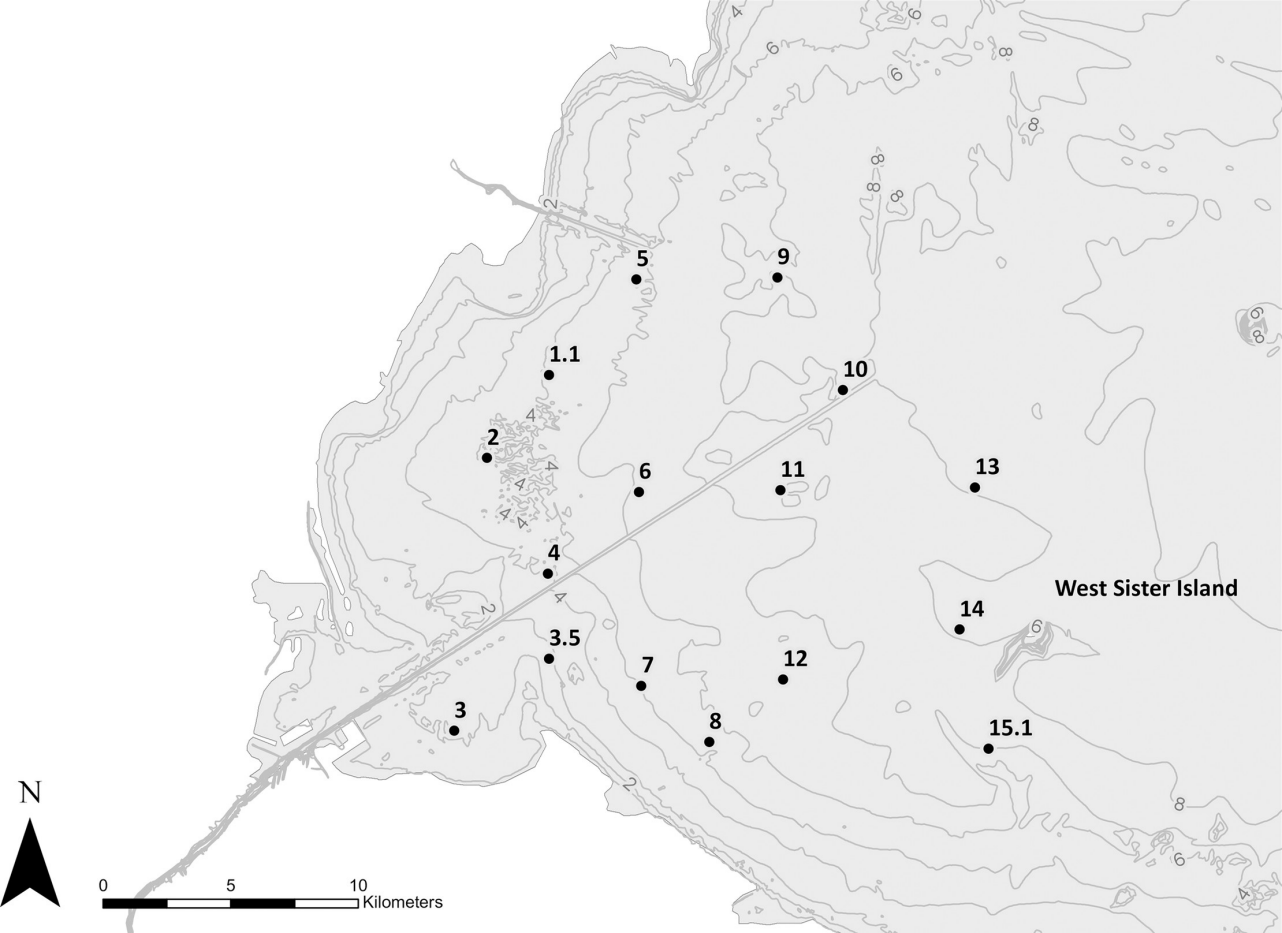
Sixteen sites were selected for sampling over a two-year period corresponding to the region within western Lake Erie where HABs are most prevalent and persistent. Bathymetry of Lake Erie (in meters) obtained from the National Geophysical Data Center, NOAA.

Lake Erie is in the southernmost portion of the Laurentian Great Lakes system, possessing a mean depth of 18.7 m, a surface area of 25,320 km^2^, and a volume of 470 km^3^ [35]. Lake Erie can be divided into 3 distinct regions: the western, central, and eastern basins. Depth gradually increases across the 3 regions, ranging from a maximum of 10 m in the western basin to 64 m in the eastern basin. Sixteen sampling sites were selected from the western basin based on the observed patterns of the predominance of past blooms (Fig 1). No provincial or federal permits or permissions were required to conduct this research as Lake Erie is a public waterbody and is not provincially nor federally protected.

Samples were collected in November 2014, April 2015, November 2015, and April 2016. Sampling times are intended to reflect conditions during early settlement (i.e. fall/November) and post-overwintering (i.e. spring/April). Most sediment samples were collected using a Pylonex HTH Sediment Corer with an inner tube diameter of 66 mm. Cores were extruded from the tube on site and the overlying water was siphoned off. The top 2 cm of sediment was removed using a sterile spatula and transferred to a sterile Whirl-Pak bag. In situations where sediment samples could not be collected using a corer, a ponar was used and sub-sampled the same top 2 cm (Table 1). Observation of all sediment samples showed that the oxidized top surface layer possessed a distinct texture and color (i.e. less compact and pale brown in color), so in instances where it was difficult to delineate the top 2 cm of ponar samples, surface sediment of these noted characteristics was collected. Furthermore, comparison of mean *Microcystis* densities and sediment organic content between ponar and corer samples always overlapped within two standard deviations when compared separately on each sampling event and pooled across all samples. Sediment samples were stored at 7° C until processed in the laboratory, within 24–48 hours of collection. In November 2014, samples were not collected for Sites 9, 10, 13, 14, and 15.1 due to weather constraints.

**Table 1:** Samples were collected at each station using either a corer or ponar. While core samples were preferable, technical difficulties or sediment composition called for the use of a ponar at several sites. Samples that were not collected are indicated by “ND” (No data).

	Collection Method			
Station ID	Nov 2014	April 2015	Nov 2015	April 2016
1.1	core	core	ponar	ponar
2	core	core	ponar	core
3	ponar	core	core & ponar	ponar
3.5	ponar	ponar	ponar	ponar
4	core	core	ponar	ponar
5	core	ponar	ponar	ponar
6	core	core	core	core
7	core	core	core	core
8	core	core	core	core
9	ND	ponar	ponar	ponar
10	ND	core	core	ponar
11	core	core	ponar	ponar
12	core	core	core	ponar
13	ND	core	core	ponar
14	ND	core	core	core
15.1	ND	core	core & ponar	core

### Laboratory sediment experiments

To confirm the viability of the *Microcystis* sediment seed stocks, a series of laboratory grow-out experiments were performed. After each sampling event, three to five grams of wet sediment was removed from a homogenized sample and added to an autoclaved 1-L flask along with 600 mL of sterile WC-Si culturing media. The WC-Si media was intended to mimic mesotrophic/eutrophic freshwater and was prepared according to the recipe described by Vanderploeg et al. [13]. Each flask (hereafter referred to as “recruitment flasks”) was corked with styrofoam and covered with aluminum foil to minimize airborne contamination but allow for gas exchange. Flasks were stored in a Percival Intellus Environmental Control biological incubator for six weeks. Temperatures were kept at 20 °C and under 12/12 day night conditions, conditions that resembled recruitment conditions in Western Lake Erie. Each flask was gently swirled weekly to minimize compaction and to ensure that cells would be exposed to the water interface as might occur at various time scales in the natural system through mixing and resuspension. Results from November 2014 culture experiments were omitted because of multiple differences in experimental design. Collected sediments were stored in Whirl-Pak bags at 7° C for several weeks prior to inoculation, sediments were inoculated in filtered lake water, and sampling occurred only after one month. Highly variable results from this first attempt led to subsequent method changes used for the remaining three sampling events.

Every two weeks and prior to swirling, 250 mL of the overlying media was siphoned from the top of each flask without disturbing the sediment to quantify cells that had recruited to the overlying water column. The siphoned water was filtered through a 3.0 μm Nucleopore Track-Etch Membrane filter and frozen at -80 °C until DNA extraction. Fresh media was added back to each flask after sub-sampling to maintain a total volume of 600 mL. This process was repeated for a total of three different time points, corresponding to t_2_ (week 2), t_4_ (week 4), and t_6_ (week 6). It was assumed that at t_0_, when the experiments were first prepared, there were no *Microcystis* cells in the overlying media.

### DNA extraction and qPCR analysis

*Microcystis* abundance in both sediments and recruitment flasks were determined using quantitative polymerase chain reaction (qPCR). Total nucleic acids were extracted from freeze-dried sediment samples using the PowerMax Soil DNA Isolation Kit (Mo Bio, CA, USA) and the provided user protocol. DNA was extracted from five grams of freeze-dried sediment barring instances of limited available sample. Total cellular nucleic acids were extracted from filtered culture samples using the Qiagen DNeasy Blood and Tissue Kit, adding a lysate homogenization step (QiaShredder spin-column) prior to DNA purification. The quantity and quality of nucleic acids were determined using a NanoDrop Lite Spectrophotometer (Thermo Scientific). DNA extract was frozen at -80 °C until analysis.

Two *Microcystis*-specific genetic targets were used during this study, the 16S rRNA gene (16S rDNA) and *mcyD* gene. Targeting the 16S rRNA gene allowed for quantification of the abundance of total *Microcystis* population. The *mcyD* gene is found within the microcystin synthetase gene operon which is responsible for the production of microcystin and is only found in potentially-toxic strains of *Microcystis* [36]. qPCR was executed using an Applied Biosystems 7500 Fast Instrument using TaqMan labeled probes (Applied Biosystems) and *Microcystis*-specific mcyD and 16S rDNA primers (Table 2). For amplification of the 16S targets, the cycling conditions were 95 °C for 10 minutes, followed by 45 cycles of 95 °C for 15 seconds and 60 °C for 1 minute. For amplification of the *mcyD* gene, the cycling conditions were 95 °C for 10 minutes, followed by 45 cycles of 95 °C for 15 seconds, 50 °C for 1 minute, and 60 °C for 1 minute. Since some *Microcystis* cells may carry multiple copies of the 16S rDNA gene and mcyD gene, data was generally expressed as “cell equivalents” [16, 37, 38].

**Table 2:** A list of primers (Integrated DNA Technologies, IA, USA) and probes (Applied Biosystems, Foster City, CA, USA) used in the qPCR analysis).

DNA Target	Primer	Sequence (5'-3')	Reference
*Microcystis* 16s rDNA	184F	GCCGCRAGGTGAAAMCTAA	[39]
	431R	AATCCAAARACCTTCCTCCC	[39]
	Probe	(Taq) FAM-AAGAGCTTGCGTCTGATTAGCTAGT-BHQ-1a	[37]
*Microcystis* mcyD	F2	GGTTCGCCTGGTCAAAGTAA	[40]
	R2	CCTCGCTAAAGAAGGGTTGA	[40]
	Probe	(Taq) FAM-ATGCTCTAATGCAGCAACGGCAAA-BHQ-1a	[37]

F: forward primer, R: reverse primer.

^a^ Black Hole Quencher-1 (quenching range 480–580 nm)

Total cell equivalents determined by qPCR were converted to cell equivalents per gram of sediment using the following equation:

$$x^{*}=\frac{x}{5}*5000*\frac{1}{y}$$,

where x* is the concentration of *Microcystis* (cell equivalents g^-1^), *x* is the total number of *Microcystis* cell copies determined by qPCR, 5 refers to the amount of extract analyzed (μL), 5000 refers to elution volume (μL), and *y* is the amount of freeze-dried sediment used for DNA extraction.

### Other sediment proxies

Chlorophyll α concentrations within sediment samples were determined by weighing 0.5–0.7 grams of wet sediment onto a Whatman GF/F filter and extracted in *N*,*N-dimethylformamide* and analyzed on a Turner Designs fluorometer [41]. Sediment phycocyanin concentrations were determined following extraction in phosphate buffer (Ricca Chemical, pH 6.8) using two freeze-thaw cycles, followed by sonication [42]. Relative fluorescence of the extracted pigments was measured on a Turner Aquaflor fluorometer. Total phosphorus content of sediments was determined using a combustion and hot HCl extraction procedure [43]. Samples were then analyzed using a SEAL AutoAnalyzer 3 HR. Particulate carbon and nitrogen were determined by flash combustion method using a Carlos Erba EA1110 configured for CHN.

### Graphs and statistics

Maps were generated in ArcMap version 10.4.1. All graphs and statistical comparisons were generated using Excel 2016 and R version 3.4.2. Kendall’s tau coefficient was calculated for pairs of continuous variables in order to determine association between those parameters. The strength of correlation coefficients were assessed based on Cohen’s standard (i.e. correlation coefficients between 0.10 and 0.29 represent a small association, coefficients between 0.30 and 0.49 represent a medium association, and coefficients of 0.50 and above represent a large association) [44]. Nonmetric multidimensional scaling was used to assess similarities between stations based on measured variables. Permutational multivariate analysis of variance (PERMANOVA) was used to test for differences in total and potentially-toxic *Microcystis* between sites and months. The PERMANOVA framework was utilized because it is readily extended to accommodate random effects, hierarchical models, mixed models, quantitative covariates, repeated measures, unbalanced and/or asymmetrical designs, and heterogeneous dispersions among groups [45]. In the event of significant pseudo-F values, pairwise PERMANOVAs were used to determine which pairs were different (alongside corrections for multiple testing). PERMANOVA outputs, pairwise testing outputs, and utilized R packages are detailed in the supplementary materials (S1 File).

## Results and discussion

**Fig 2:**
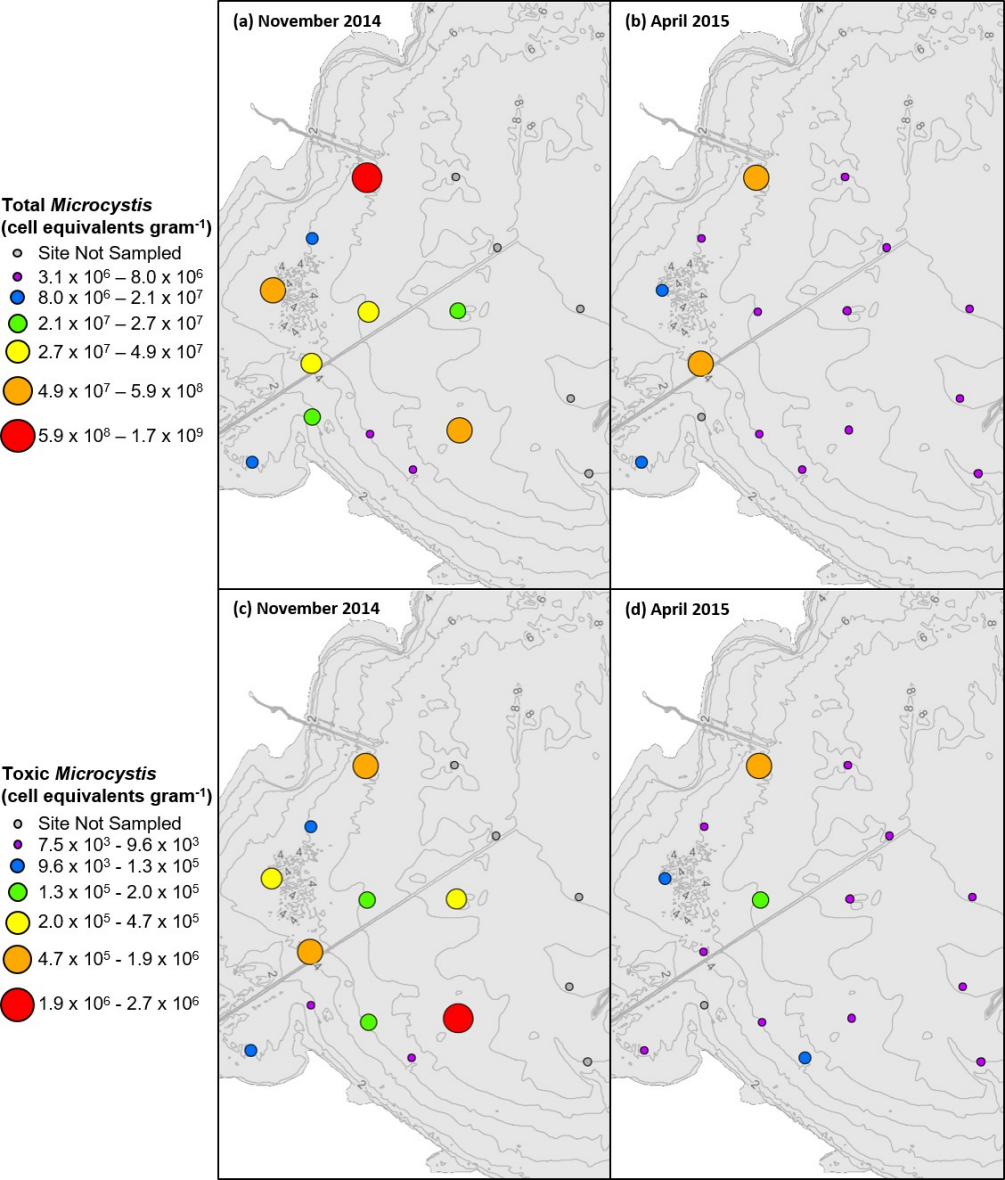
**Total *Microcystis* cell equivalents in (a) November 2014 compared to (b) April 2015 per gram dry sediment and potentially-toxic *Microcystis* cell equivalents in (c) November 2014 and (d) April 2015 per gram dry sediment.** Ranges for each category selected based on ArcGIS quantile classification of November 2014 data.

**Fig 3:**
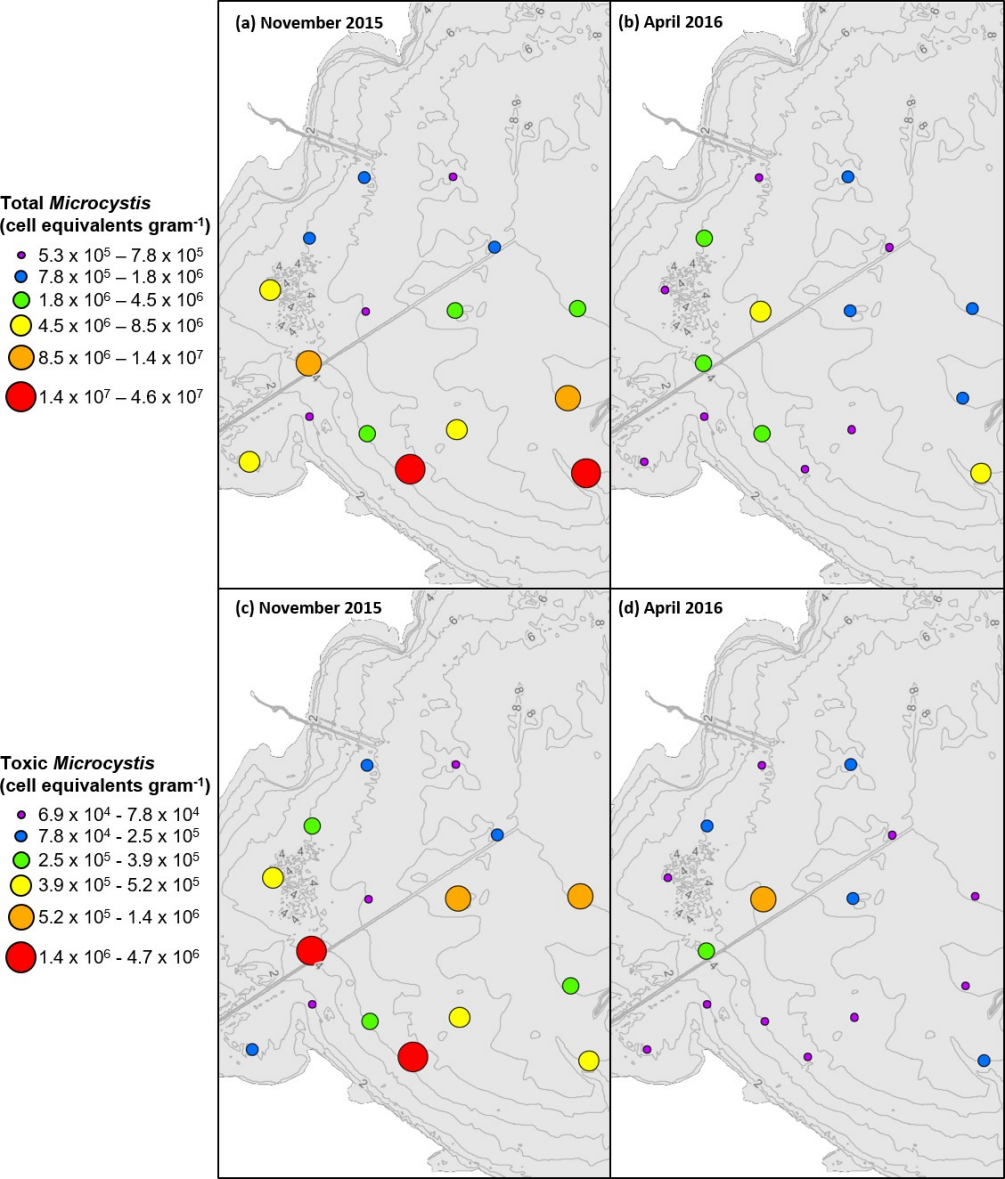
**Total *Microcystis* cell equivalents in (a) November 2015 compared to (b) April 2016 per gram dry sediment and potentially-toxic *Microcystis* cell equivalents in (c) November 2015 and (d) April 2016 per gram dry sediment.** Ranges for each category selected based on ArcGIS quantile classification of November 2015 data.

In both sampling years, total *Microcystis* cell equivalents within the surface sediment decreased from November to April by at least one order of magnitude (Table 3). The observed mortality of cells across the overwintering period was consistent with the results of previous studies [21–26]. The basin-wide average of the over-winter decline in total (77%) and potentially-toxic (90%) cell abundance was similar in both years. One inconsistent result was that the basin-wide average abundance of total *Microcystis* was considerably greater in November 2014 compared to subsequent sampling periods (Figs 2 and 3). The 10-fold greater concentration was greatly influenced by one value (Site 5), and when excluded the average reduced more than half to 1100 cell equivalents per gram. The highest concentrations of *Microcystis* were not found closest to the river mouth, but there was a slight tendency for concentrations to be higher closer to shore and in relatively shallow water.

**Table 3:** Basin-wide averages of measured parameters for each seasonal sampling (Mean ± SD).

	Total (10^5^ cell equivalents/g)	Potentially-Toxic (10^5^ cell equivalents/g)	CHLA (μg/g)	PC (μg/g)	TP (μg P/g)	PON (μg N/g)	POC (μg C/g)
Nov-14	2600 ± 5100	6.6 ± 9.1	7.0 ± 9.5	0.16 ± 0.07	0.87 ± 0.17	2.6 ± 0.9	37 ± 5.3
Apr-15	260 ± 650	0.78 ± 1.3	3.9 ± 1.9	0.18 ± 0.19	0.83 ± 0.18	2.2 ± 0.7	33 ± 8.6
Nov-15	81 ± 120	8.6 ± 14	6.4 ± 2.9	0.35 ± 0.11	0.70 ± 0.18	2.4 ± 1.0	33 ± 11
Apr-16	19 ± 24	0.9 ± 1.4	5.0 ± 2.0	0.27 ± 0.07	0.71 ± 0.21	2.1 ± 1.0	32 ± 8.1

CHLA: chlorophyll α, PC: phycocyanin, TP: total phosphorus, PON: particulate organic nitrogen, POC: particulate organic carbon

Somewhat unexpectedly, the average abundance of total sediment *Microcystis* in November 2015 was not elevated despite a record-level bloom season in both abundance and extent throughout the summer of 2015. The average annual bloom in 2015 had a cyanobacteria index (CI) value of 5 while the average annual bloom in 2014 had a CI value of 1.5, where one CI equals 10^20^ cells [46]. Despite the much higher bloom, yet the average abundance of total *Microcystis* in the sediment was substantially lower in November 2015 relative to November 2014. Sediment organic content and pigment concentrations were also not elevated in fall 2015 relative to fall 2014 and may indicate a dilution effect from the high tributary sediment load which was 50% higher in 2015 compared to 2014 (Heidelberg College, https://ncwqr.org/monitoring). Scatter plots detailing specific data points for chlorophyll α, phycocyanin, total phosphorus, particulate organic nitrogen, and particulate organic carbon can be found in the supplemental materials (S1 Fig, S2 Fig, S3 Fig, S4 Fig and S5 Fig, respectively).

**Fig 4:**
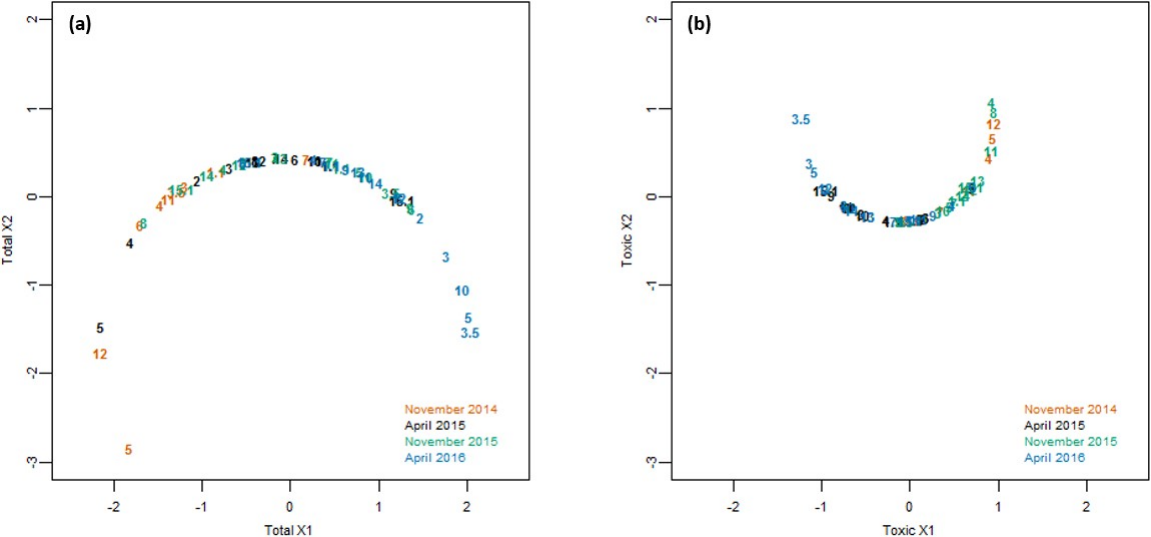
Nonmetric multidimensional scaling of data. Non-Euclidean Bray-Curtis distances were calculated based on the following variables: Depth (m), Distance from the Maumee River (km), Distance from the Detroit River (km), chlorophyll α (μg g^-1^), phycocyanin (μg g^-1^), PON (μg N g^-1^), POC (μg C g^-1^), TP (μg P g^-1^), and (a) total *Microcystis* (cell equivalents g^-1^) or (b) potentially-toxic *Microcystis* (cell equivalents g^-1^).

Nonmetric multidimensional scaling of data revealed no clear patterns of dissimilarity among stations but did show some dissimilarity among sampling events (Fig 4). Observations seen in Fig 4 are corroborated by subsequent PERMANOVA analyses of the same distance matrixes portrayed in Fig 4 (Table 4). Ensuing pairwise comparisons showed that overall total *Microcystis* in November 2014 was greater than that of subsequent sampling events. However, total *Microcystis* in November 2015 was similar to the two April events. Dissimilarity was detected in potentially-toxic *Microcystis* between November 2015 and November 2014 as well as April 2015 and April 2016, but remaining pairs were not significantly dissimilar. Overall, while there was a significant difference in the abundance of total *Microcystis* and potentially-toxic *Microcystis* between some seasons and years, there was no difference between the abundance of *Microcystis* at different sites.

**Table 4:** PERMANOVA tests for total and potentially-toxic *Microcystis* (cell equivalents g^-1^) between sites (1.1–15.1) and event (November 2014, April 2015, November 2015, and April 2016). Tests were based on the non-Euclidean Bray-Curtis dissimilarity measure and were done using 999 permutations under the reduced model. Bold values indicate significant differences at p <0.05.

*Microcystis spp*.	Source	df	SS	R^2^	Pseudo-F	Pr (>F)
Total	Site	15	3.558	0.25791	1.1489	0.261
	Event	3	2.3919	0.17338	3.8617	**0.002**
	Residual	38	7.8457	0.56871	-	-
	Total	56	13.7957	1	-	-
Potentially-toxic	Site	15	2.5099	0.18552	0.8146	0.783
	Event	3	3.214	0.23756	5.2157	**0.001**
	Residual	38	7.8053	0.57693	-	-
	Total	56	13.5291	1	-	-

**Fig 5:**
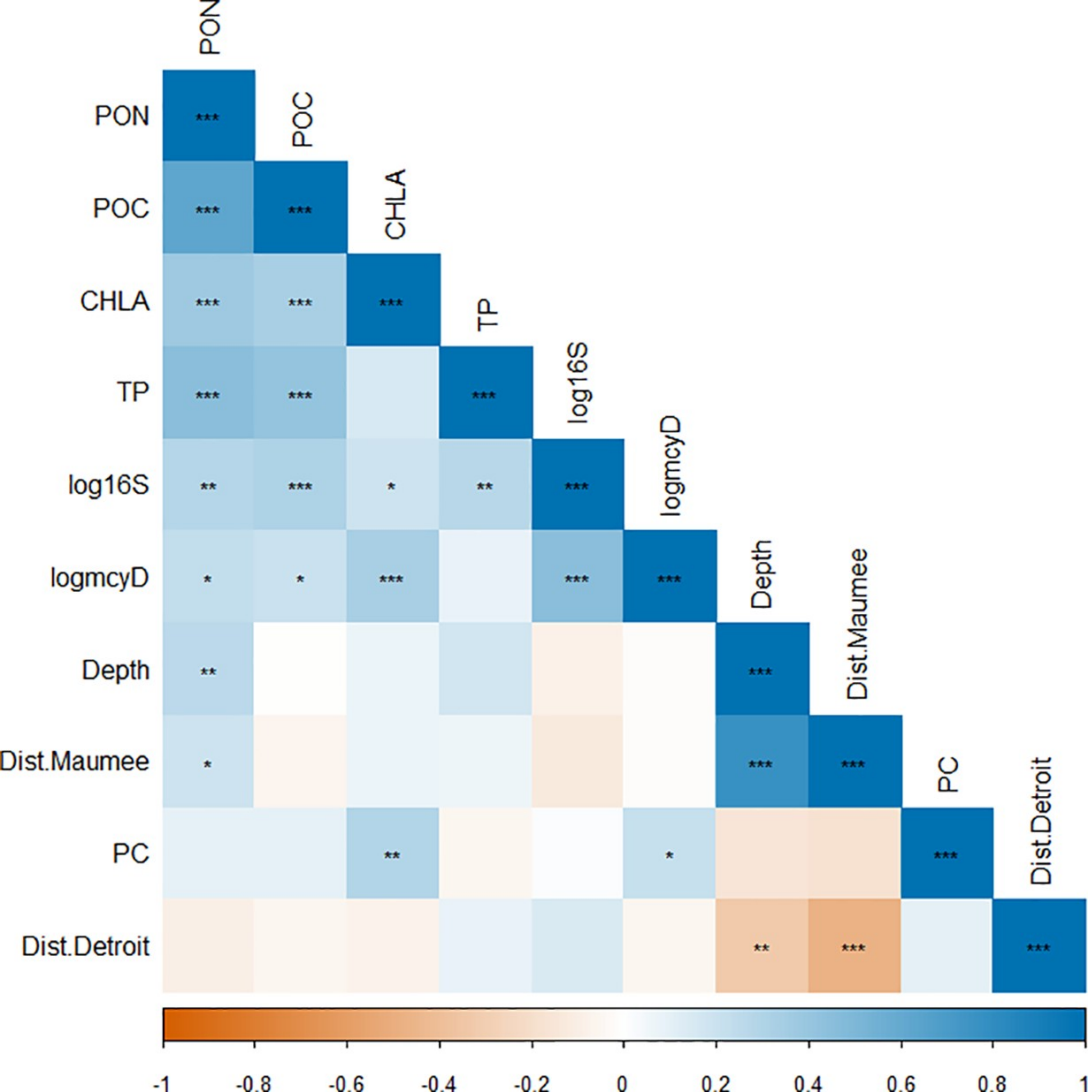
Correlation matrix of various parameters measured in the study. Values of correlation coefficients are indicated by color and statistical significance of correlations are indicated by symbol (*: p<0.05, **: p<0.01, ***: p<0.001). Both total *Microcystis* (cell equivalents g^-1^) and potentially-toxic *Microcystis* (cell equivalents g^-1^) were log-transformed. The strength of correlation coefficients were assessed based on Cohen’s standard (i.e. correlation coefficients between 0.10 and 0.29 represent a weak association, coefficients between 0.30 and 0.49 represent a moderate association, and coefficients of 0.50 and above represent a strong association).

Looking at bivariate plots of various parameters, log-transformed data for total and potentially-toxic *Microcystis* are strongly associated with one another, but neither parameter is strongly associated with sediment organic content or pigment concentrations (S6 Fig). A similar lack in association between total *Microcystis* and potentially-toxic *Microcystis* and other measured parameters is seen when comparing calculated Kendall’s Tau coefficients between continuous parameters (Fig 5). In contrast to previous studies which found that the accumulation of total *Microcystis* in sediments was associated with both depth and distance offshore [21, 22, 24], neither of these variables were highly associated with total *Microcystis*. These differences may result from the fact that the previous studies compared shallow depths (~0–6 m) to deeper depths (>20m), whereas the range of depths in the present study (3–9 m) was not great enough to observe significant differences in *Microcystis* abundance. Curiously, the log of potentially-toxic *Microcystis* was moderately associated with chlorophyll α concentration whereas total *Microcystis* was weakly associated with chlorophyll α concentration. The absence of a large correlation between *Microcystis*, pigments, and nutrients could be due to the influence of other overwintering phytoplankton species or the mixing of allochthonous sediments with that of lake derived organic matter that has accumulated in the sediment [47–50]. The lack of a strong association between both total and potentially-toxic *Microcystis* abundance and the other parameters suggests that commonly used proxies for estimating *Microcystis* cell abundance in the water column are not appropriate for estimating cell abundance in the sediments.

### Lab sediment experiment results

**Fig 6:**
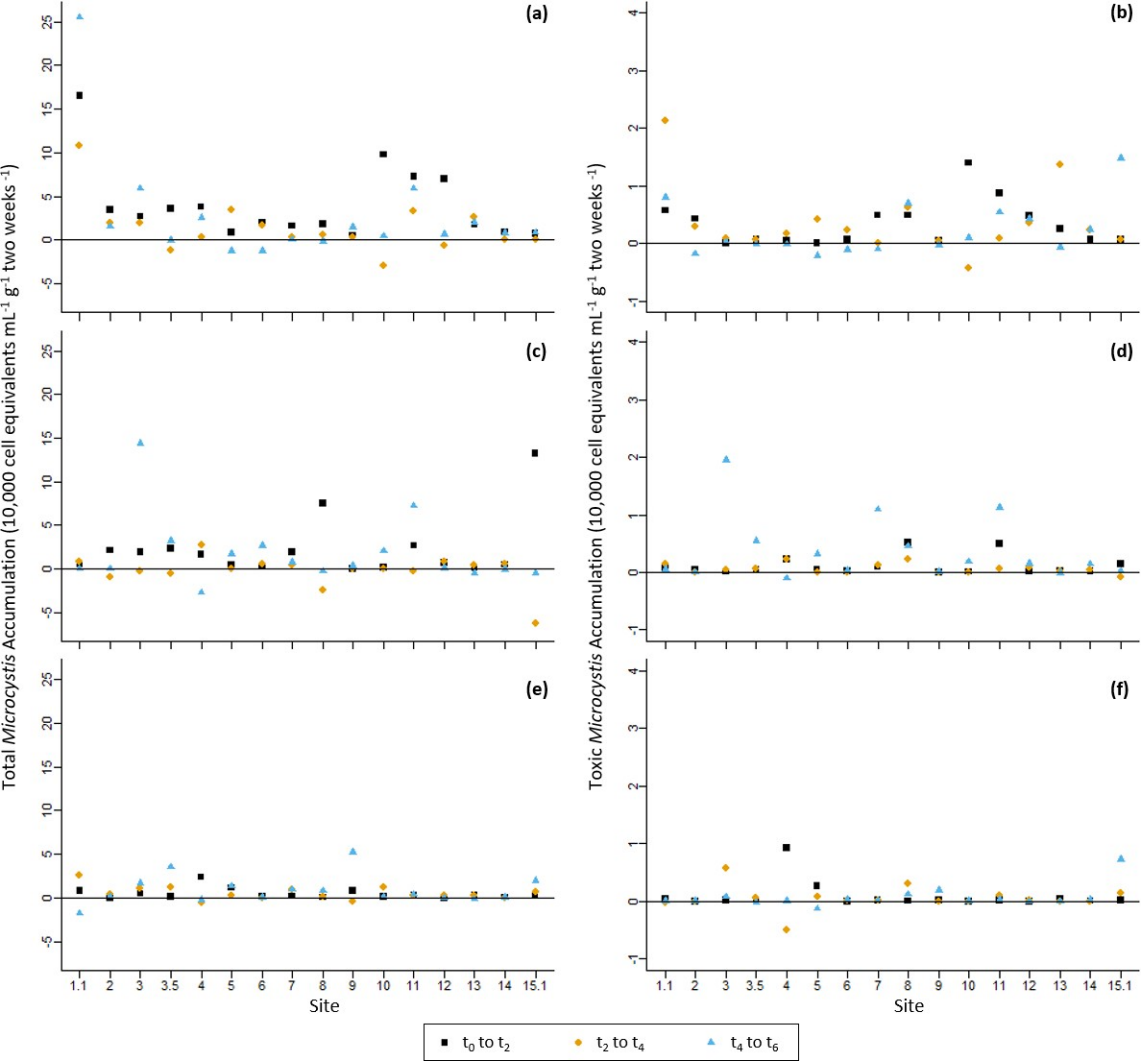
**Log-transformed accumulation data for April 2015 cultures for a) total *Microcystis* and b) potentially-toxic *Microcystis*, November 2015 cultures for c) total *Microcystis* and d) potentially-toxic *Microcystis*, and April 2016 cultures for e) total *Microcystis* and f) potentially-toxic *Microcystis*, all in terms of change in cell equivalents mL**^**-1**^ **g**^**-1**^ **2 weeks**^**-1**^. Squares represent accumulation from t_0_ to t_2_, diamonds represent accumulation from t_2_ to t_4_, and triangles represent accumulation from t_4_ to t_6_.

In the grow-out experiments, both potentially-toxic and non-toxic cells recruited to the overlying media in every recruitment flask, however, cell accumulation over time varied substantially between flasks (Fig 6). Some recruitment flasks experienced continuous positive accumulation in *Microcystis* over the 6-week incubation period while other flasks had initial spikes in total *Microcystis* densities, followed by constant or decreased concentrations over the remainder of the incubation period. Some of the variability may have been due to variability in subsequent growth following initial recruitment or failure to maintain position within the overlying media. These observations are reflected in the PERMANOVA analysis of cell accumulation data (Table 5). Results for total *Microcystis* accumulation were similar across sites, sampling event, and time intervals. However, dissimilarity was detected between time intervals for potentially-toxic *Microcystis* accumulation. Subsequent pairwise analysis showed that accumulation from t_0_ to t_2_ was significantly higher than the other two measured time intervals. Despite variability in accumulation rates, an increase abundance of cells was seen in all cultures relative to t_0_, affirming the viability of the overwintering cells in the lake sediments.

**Table 5:** PERMANOVA tests for both total and potentially-toxic *Microcystis* accumulation in cultures (cell equivalents mL^-1^ g^-1^ 2 weeks^-1^) between sites (1.1–15.1), event (November 2014, April 2015, November 2015, and April 2016), and period (t_0_ to t_2_, t_2_ to t_4_, and t_4_ to t_6_). Tests were based on the non-Euclidean Bray-Curtis dissimilarity measure and were done using 999 permutations under the reduced model. Bold values indicate significant differences at p <0.05.

*Microcystis spp*.	Source	df	SS	R^2^	Psuedo-F	Pr (>F)
Total	Event	2	27906	0.85913	46.9555	0.182
	Site	15	31317	0.96415	7.0261	0.385
	Period	2	-63589	-1.95767	-106.9962	0.815
	Residual	124	36847	1.13439	-	-
	Total	143	32482	1	-	-
Potentially-toxic	Event	2	-70740	-1.56556	-154.026	0.964
	Site	15	45060	0.99724	13.082	0.057
	Period	2	42389	0.93813	92.297	**0.044**
	Residual	124	28475	0.63019	-	-
	Total	143	45185	1	-	-

**Fig 7:**
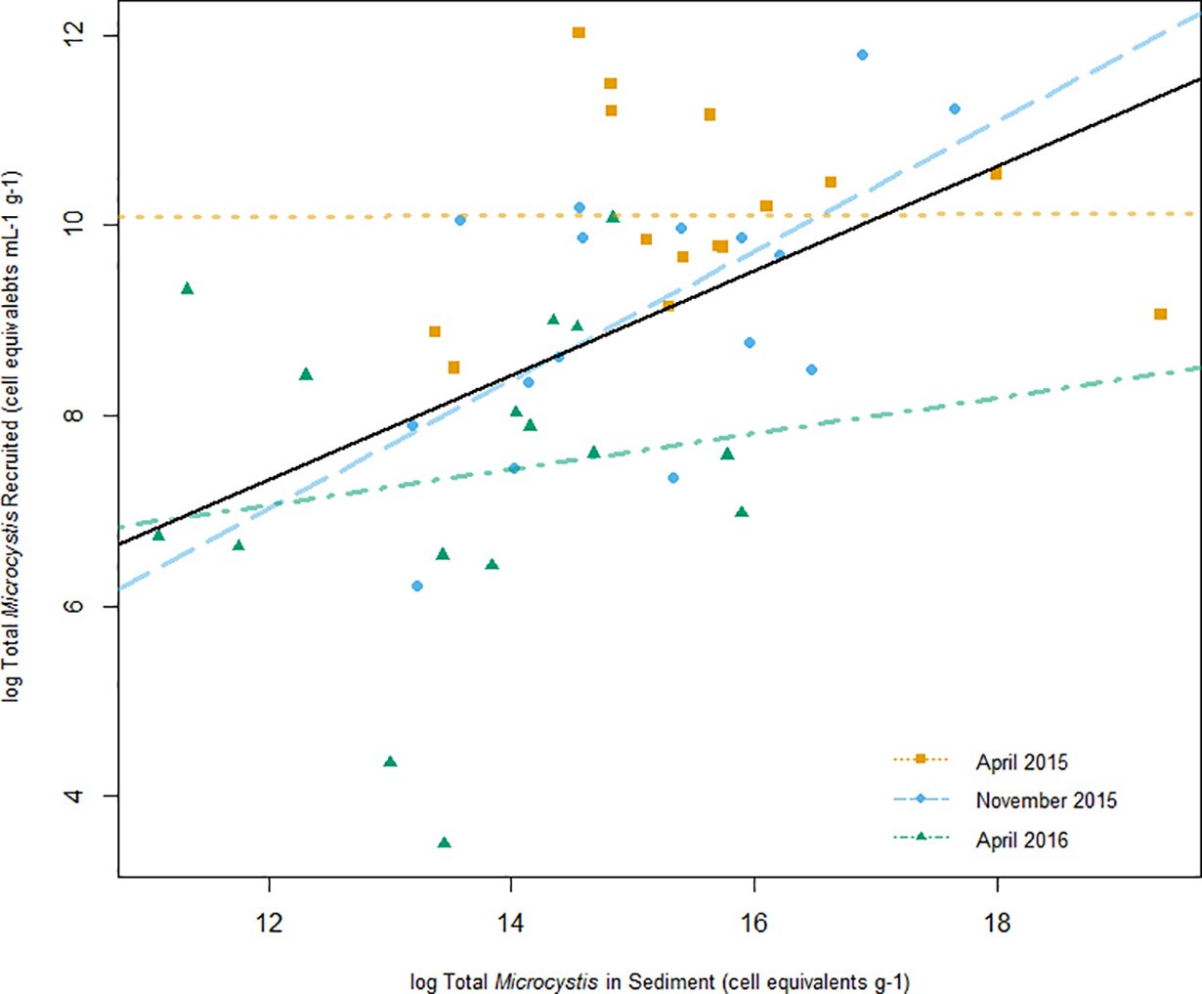
*Microcystis* vitality plotted against *Microcystis* abundance. The amount of total *Microcystis* in overlying water at t_2_ (log cell equivalents mL^-1^ g^-1^) plotted against *Microcystis* sediment abundance (log cell equivalents g^-1^). Values at t_2_ were used to minimize the impacts of sampling dilution and influences of growth. The plot shows simple linear models for April 2015 data (dotted line), November 2015 data (dashed line), April 2016 data (dot-dashed line), and for overall data (solid line).

When comparing the quantity of total *Microcystis* in field sediments versus the recruitment flasks, which are assumed to represent the abundance and viability of sediment seed stocks, respectively, the sediment samples with the highest abundance were not always the ones with the greatest viability. For example, in April 2015, sediments for Sites 5 (6.4 x 10^5^ ± 2.7 x 10^5^ cell equivalents g^-1^) and 15.1 (2.5 x 10^8^ ± 7.4 x 10^7^cell equivalents g^-1^) had the lowest and greatest total abundances, respectively; whereas both of the corresponding recruitment flasks yielded similarly low quantities of total *Microcystis* throughout the incubation period (7.9 x 10^3^ ± 9.9 x 10^3^ cell equivalents ml^-1^ g^-1^ and 1.1 x 10^4^ ± 1.5 x 10^3^ cell equivalents ml^-1^ g^-1^, respectively, by t_6_). In fact, the recruitment flask that yielded the greatest quantity of total *Microcystis* accumulation was from Site 1.1 sediments (3.6 x 10^5^ ± 2.0 x 10^4^ cell equivalents ml^-1^ g^-1^), which had a relatively low initial abundance compared to many of the sites. Linear regressions were run on log-transformed data by experiment and overall data (Fig 7). While models for April 2015 and April 2016 data were not significant (April 2015 Adjusted R^2^ = -0.08, p = 0.98; April 2016 Adjusted R^2^ = -0.04505, p = 0.56), models for November 2015 and overall data were significant (November 2015 Adjusted R^2^ = 0.3777, p = 0.01; Overall Adjusted R^2^ = 0.228, p = 4.1 x 10^−4^). These results suggest that factors that control the abundance of *Microcystis* at a given location do not strongly influence the vitality of surviving, over-wintering vegetative seed stocks. As mentioned previously, the variance observed in the grow-out experiments could also be influenced by recruited cells not able to maintain buoyancy within the water column.

The percentage of potentially-toxic *Microcystis* in the sediment versus the recruitment flasks indicates that potentially-toxic strains of *Microcystis* may more readily recruit into the water column compared to non-toxic strains (Table 6 & S7 Fig). In the sediment, potentially-toxic strains made up 1%, 16%, and 5% of the benthic populations on average in April 2015, November 2015, and April 2016, respectively. In the corresponding recruitment flasks using these sediments, the potentially-toxic strains made up 17%, 14%, and 12% of the total population on average, respectively. PERMANOVA results and subsequent pairwise comparisons for the percentage of potentially-toxic *Microcystis* also indicate that the percent potentially-toxic *Microcystis* at t_2_ is different from the percentage at both t_4_ and t_6_ (though there was not a significant dissimilarity between t_4_ and t_6_) (Table 7). Pairwise comparisons showed that, while the percentage of potentially-toxic *Microcystis* was similar between the cultures from the two April events, the cultures from the November sampling event were dissimilar from the two April events. Finally, pairwise comparisons showed that the percent of potentially-toxic *Microcystis* in the sediments were dissimilar between all three time points.

**Table 6:** Average percentage of total *Microcystis* population that was potentially-toxic in both the sediments and the recruitment flasks.

		Recruitment Flasks			
	Basin-Wide	t_2_	t_4_	t_6_	Overall
Apr-15	1%	10%	20%	22%	17%
Nov-15	16%	8%	12%	22%	14%
Apr-16	5%	9%	13%	14%	12%

**Table 7:** PERMANOVA of percentage of potentially-toxic *Microcystis* based on sites (1.1–15.1), event (November 2014, April 2015, November 2015, and April 2016), and time point (initial sediment, t_2_, t_4_, t_6_). Tests were based on the non-Euclidean Bray-Curtis dissimilarity measure and were done using 999 permutations under the reduced model. Bold values indicate significant differences at p <0.05.

	Source	df	SS	R^2^	Psuedo-F	Pr (>F)
Percent-Potentially Toxic	Event	2	1.278	0.03392	3.5474	**0.005**
	Site	15	3.583	0.09513	1.3265	0.105
	Time	3	2.550	0.06769	4.7194	**0.001**
	Residual	168	30.256	0.80325	-	-
	Total	188	37.667	1	-	-

The shift in community composition towards a greater proportion of potentially-toxic strains in the water column relative to the sediment seed stocks suggests one of two things: a) that those strains recruit more readily than non-toxic strains or b) both strains are recruited at similar rates, but potentially-toxic strains grow at faster rates than non-toxic strains once in the water column. In general these results are consistent with observations from weekly monitoring results by NOAA GLERL covering the same region which found that the percentage of potentially-toxic cells is highest during the early stages of the bloom and declines throughout the summer [18]. Other temperate systems have shown that the proportion of toxic genotypes are higher during the early portion of blooms, but also suggest other environmental conditions that influence growth conditions beyond seasonal influences [29, 51].

### Potential contributions to annual algal blooms

Results from the present study provide some additional insight regarding whether sediment recruitment is relevant to bloom initiation, growth, and spatial extent of HABs in Western Lake Erie. Comparisons based simply on initial overwintering cell abundance cannot fully explain the rate at which subsequent blooms develop. However, coupling our abundance estimates along with potential recruitment rates and conservative growth rates indicates the potential importance of sediment inocula to algal bloom initiation.

For example, based on 2015 NOAA GLERL weekly monitoring data within a similar area of western Lake Erie, an average increase of 30 μg/L chlorophyll α and 11 μg/L phycocyanin was observed over a 1-week period at the end of July, signaling a rapid initiation of a cyanobacteria bloom. Assuming an average water column depth of 7 m and an area of 375 km^2^ (i.e. the size of an area that encapsulates all the site locations in this study; S6 Fig), an increase in 30 μg/L chlorophyll α corresponds to ~79 MT of chlorophyll α. Assuming the cyanobacteria bloom is largely comprised of *Microcystis* and using known values for the average chlorophyll α content of *Microcystis* cells in Western Lake Erie [52], 79 MT of chlorophyll α equates to 2.7 x 10^20^ cells or 1.02 x 10^5^ cells mL^-1^ (S2 File). Based on total *Microcystis* sediment abundance data from the April 2015 samples collected for this study, the average density of *Microcystis* in the sediment was 9.89 x 10^6^ cell equivalents g^-1^. Based on an assumption that the mixed surface layer exposed to the water interface extends to the top 0.5 cm of the surface sediment, an estimate of the total *Microcystis* cells that could re-enter the water column is 6.40 x 10^3^ cell equivalents mL^-1^, which would only account for 6% of the weekly increase. Assuming these introduced cells grow at a conservative growth rate of 0.27 days^-1^ for 1 week [53], cell density would increase to 4.24 x 10^4^ cells mL^-1^, which accounts for 42% of the calculated weekly increase based on observed change in chlorophyll α.

While the introduction of sediment *Microcystis* alone can only explain 6% of the rapid weekly increase in *Microcystis* cells in the example, pairing sediment abundances with a conservative growth rate can explain approximately half of the increase in water column populations in the summer. The magnitude of this potential contribution of overwintering cells to bloom initiation and the large spatial extent over which blooms rapidly develop provide some evidence for the importance of this sediment recruitment process for subsequent bloom development. It is unlikely that seeding from riverine input or growth from extremely rare concentrations in the water column during transition to summer conditions can fully explain the spatial and temporal scales over which blooms develop. Of course, these calculations do not take into consideration the many processes that disrupt or enhance growth rates in Western Lake Erie. It also assumes that all the sediment *Microcystis* that can be recruited is done so nearly simultaneously and that growth initiates immediately. We also recognize that cells may be settling and recruiting multiple times throughout the bloom season. Therefore, these calculations should only be used to evaluate the *potential* contribution of sediment recruitment to bloom initiation and not the absolute contribution.

## Conclusions

Overall, this study provides further evidence that summer blooms in Western Lake Erie could, at least partially, be seeded internally from lake sediments. Future studies should seek to analyze sediments throughout the entire bloom life cycle (i.e. overwintering period, spring recruitment, summer blooms, and fall settling) to better comprehend the role of sediment populations throughout the entire cycle of bloom development since it is unknown whether populations are settling and re-seeding on multiple occurrences. If such a process was happening it would provide a mechanism to enhance or extend the duration of bloom, particularly if settled cells could take advance of large pools of nutrients associated with the sediments.

## Supporting information

S1 Fig Scatterplot of chlorophyll α data across all sampling events. (TIF)

S2 Fig Scatterplot of phycocyanin data across all sampling events. (TIF)

S3 Fig Scatterplot of total phosphorus data across all sampling events. (TIF)

S4 Fig Scatterplot of particulate organic nitrogen data across all sampling events. (TIF)

S5 Fig Scatterplot of particulate organic carbon data across all sampling events. (TIF)

S6 Fig Scatterplot matrix of various parameters measured in the study. Both total *Microcystis* (cell equivalents g^-1^) and potentially-toxic *Microcystis* (cell equivalents g^-1^) were log-transformed. While included in all data analyses, a single outlier for chlorophyll α was removed from the plot for clarity of data presentation.
(TIF)

S7 Fig Percentage of potentially-toxic *Microcystis* in both the initial sediment and all three culture time points for (top) April 2015, (middle) November 2015, and (bottom) April 2016. (TIF)

S8 Fig Working area for discussion analysis. To estimate the potential contribution of *Microcystis* sediment recruitment to the average annual bloom, a theoretical area was established to enable quantitative comparisons between benthic and pelagic populations of *Microcystis*. Since values for variables and constants are developed based on the 16 sites analyzed in this study, an area encapsulating those sites was used for this analysis. The area is ~375 km^2^ and, assuming an average depth of 7 m, contains a water volume of 2.625 x 10^12^ L.
(TIF)

S1 File Metadata of R code including dataset manipulations, distance matrix generation, PERMANOVA outputs, pairwise comparison outputs, and relevant R packages. (DOCX)

S2 File Additional calculations used to determine potential impacts of harmful algal blooms. (DOCX)

S3 File Data for sampling events including total *Microcystis*, potentially-toxic *Microcystis*, and other measured parameters. Referred to as “RDataSummary” in R code.
(TXT)

S4 File Data from grow-out experiments including total and potentially-toxic *Microcystis* at various time points. Referred to as “RDataCulture” in R code.
(TXT)

S5 File Culture accumulation rates from grow-out experiments. Referred to as “RDataCultureAccumulation” in R code.
(TXT)
