# Supplementary figures and images for: Establishing spatial and temporal patterns in Microcystis sediment seed stock viability and their relationship to subsequent bloom development in Western Lake Erie
Source: PLoS One. 2018 Nov 21;13(11):e0206821. doi: 10.1371/journal.pone.0206821 (PMC6248936; doi:10.1371/journal.pone.0206821)

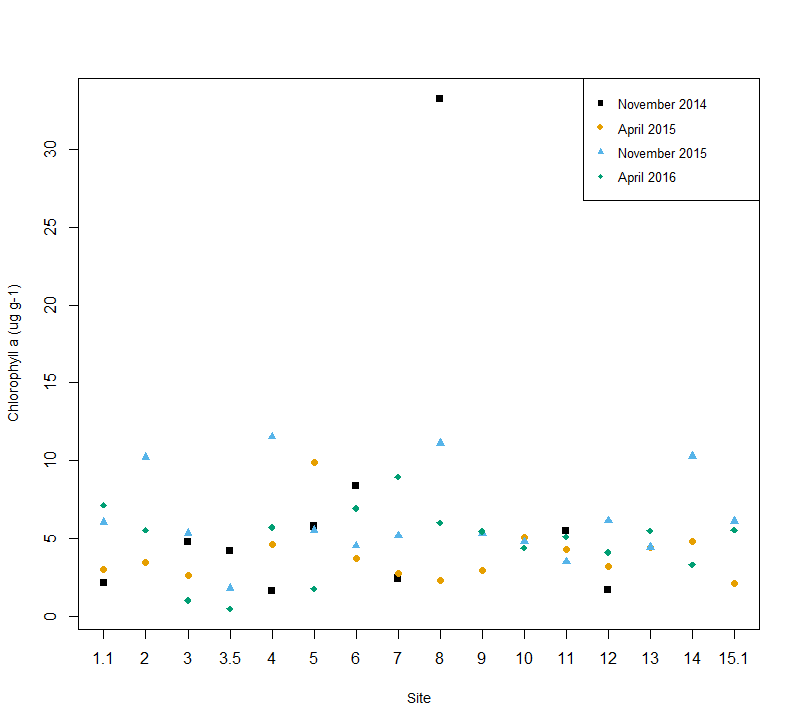

Supplement: S1 Fig — (TIF) [file pone.0206821.s001.tif]

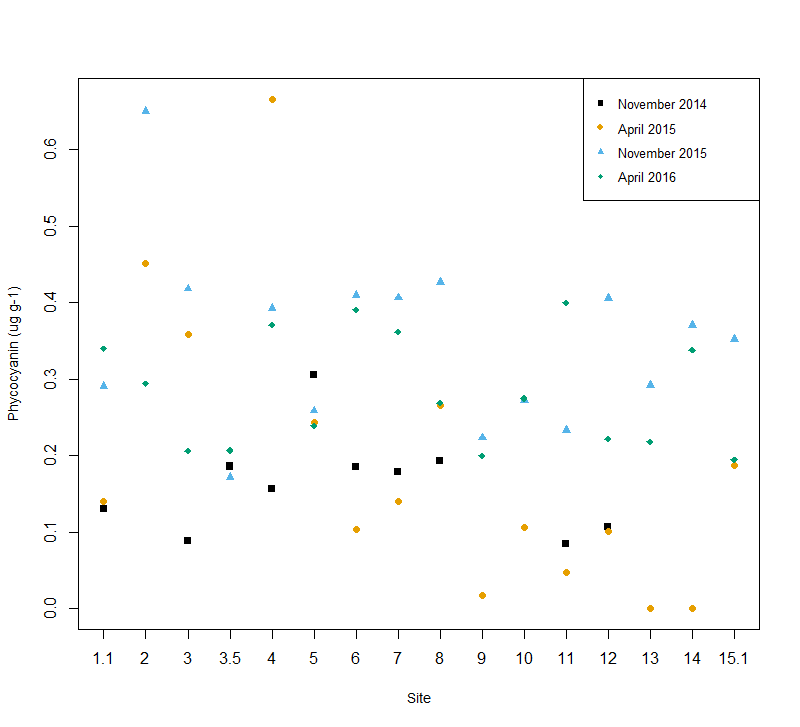

Supplement: S2 Fig — (TIF) [file pone.0206821.s002.tif]

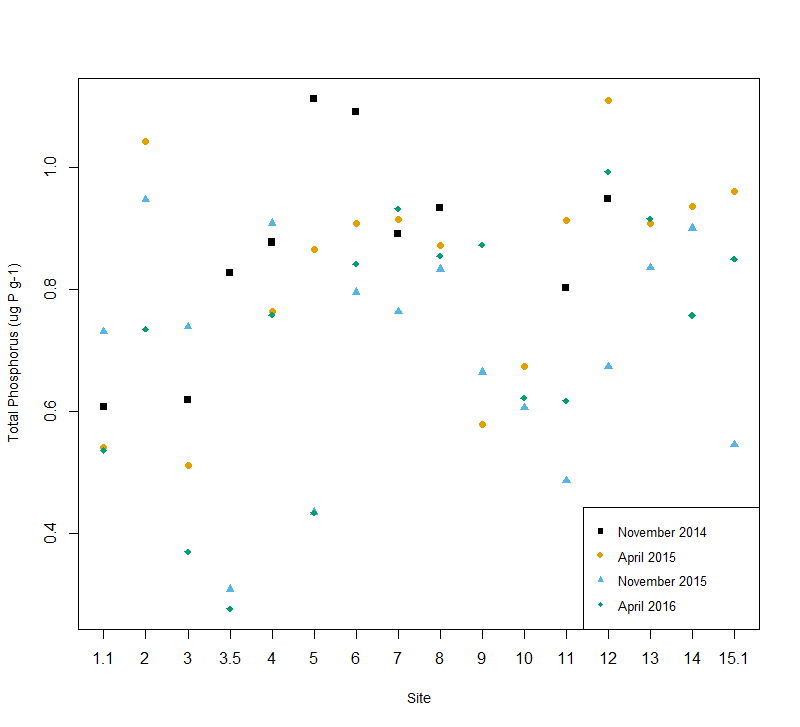

Supplement: S3 Fig — (TIF) [file pone.0206821.s003.tif]

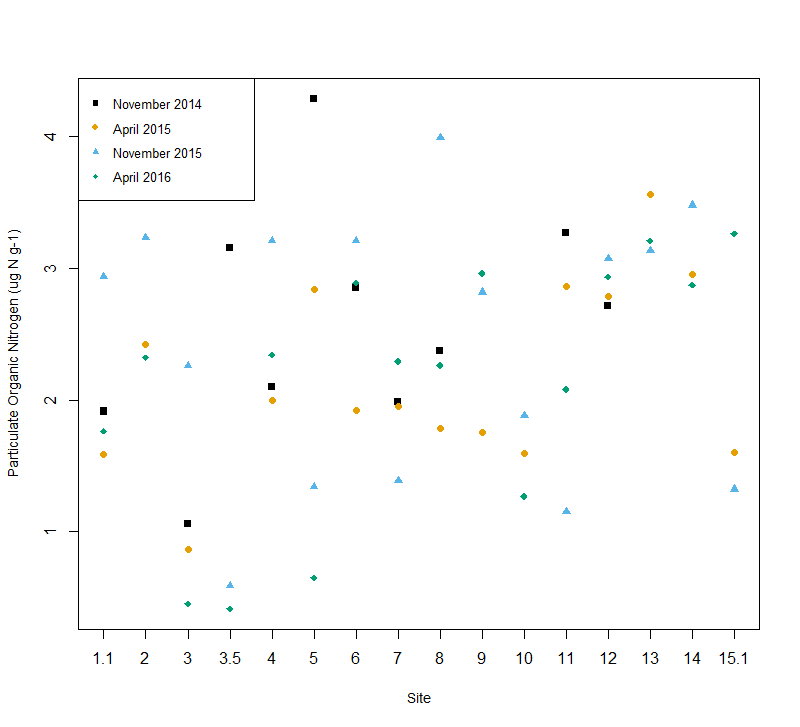

Supplement: S4 Fig — (TIF) [file pone.0206821.s004.tif]

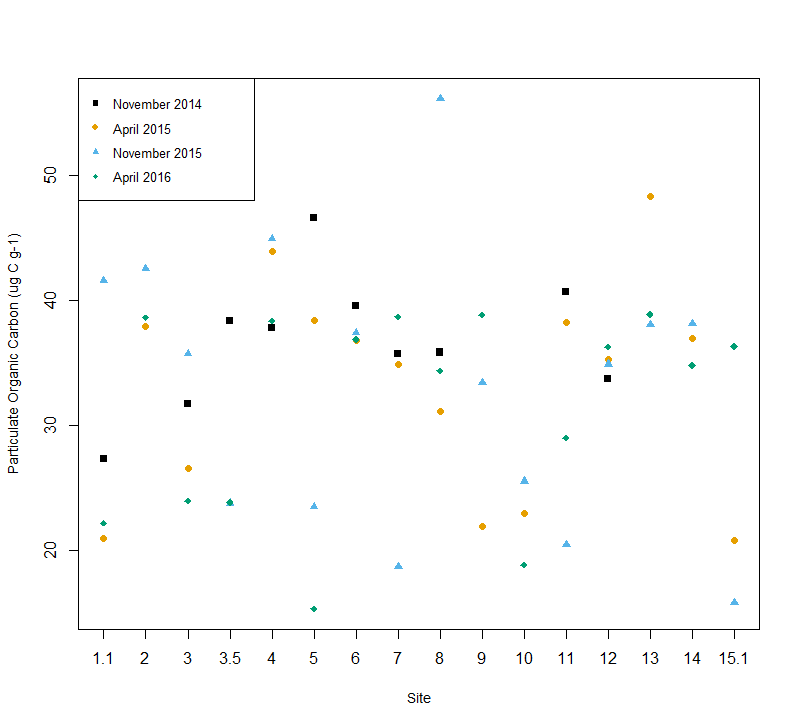

Supplement: S5 Fig — (TIF) [file pone.0206821.s005.tif]

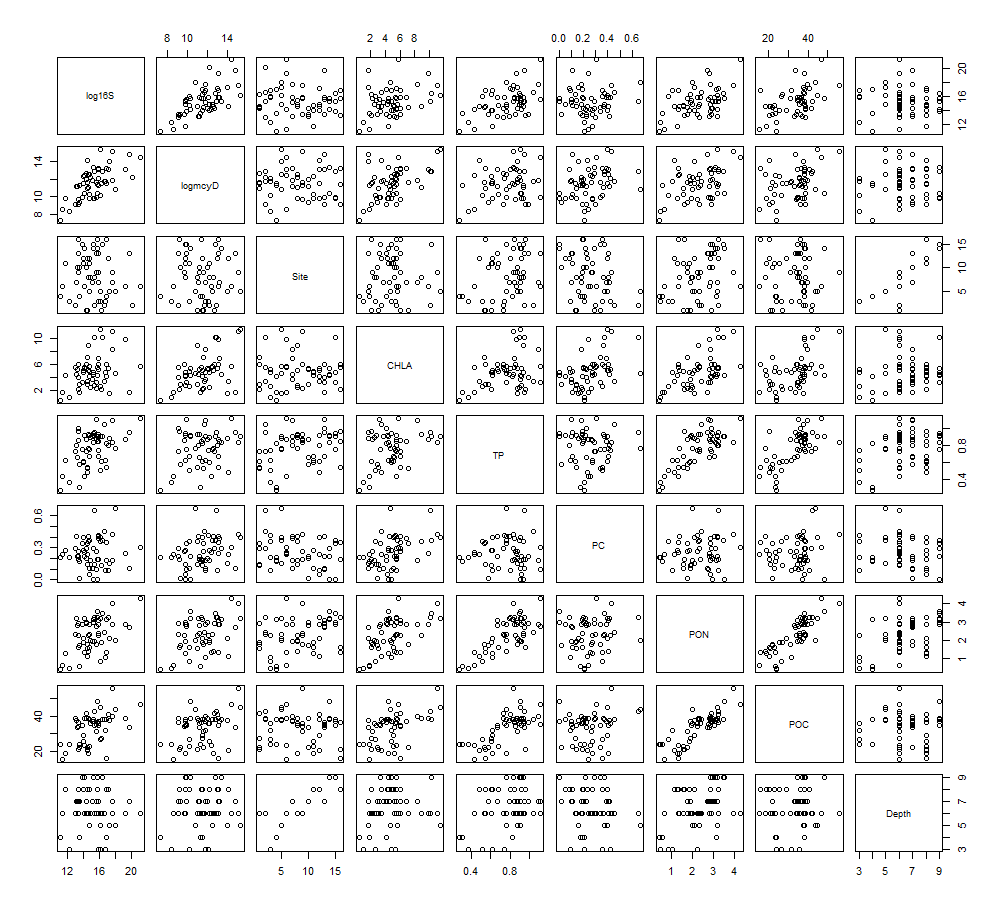

Supplement: S6 Fig — Both total Microcystis (cell equivalents g-1) and potentially-toxic Microcystis (cell equivalents g-1) were log-transformed. While included in all data analyses, a single outlier for chlorophyll α was removed from the plot for clarity of data presentation. (TIF) [file pone.0206821.s006.tif]

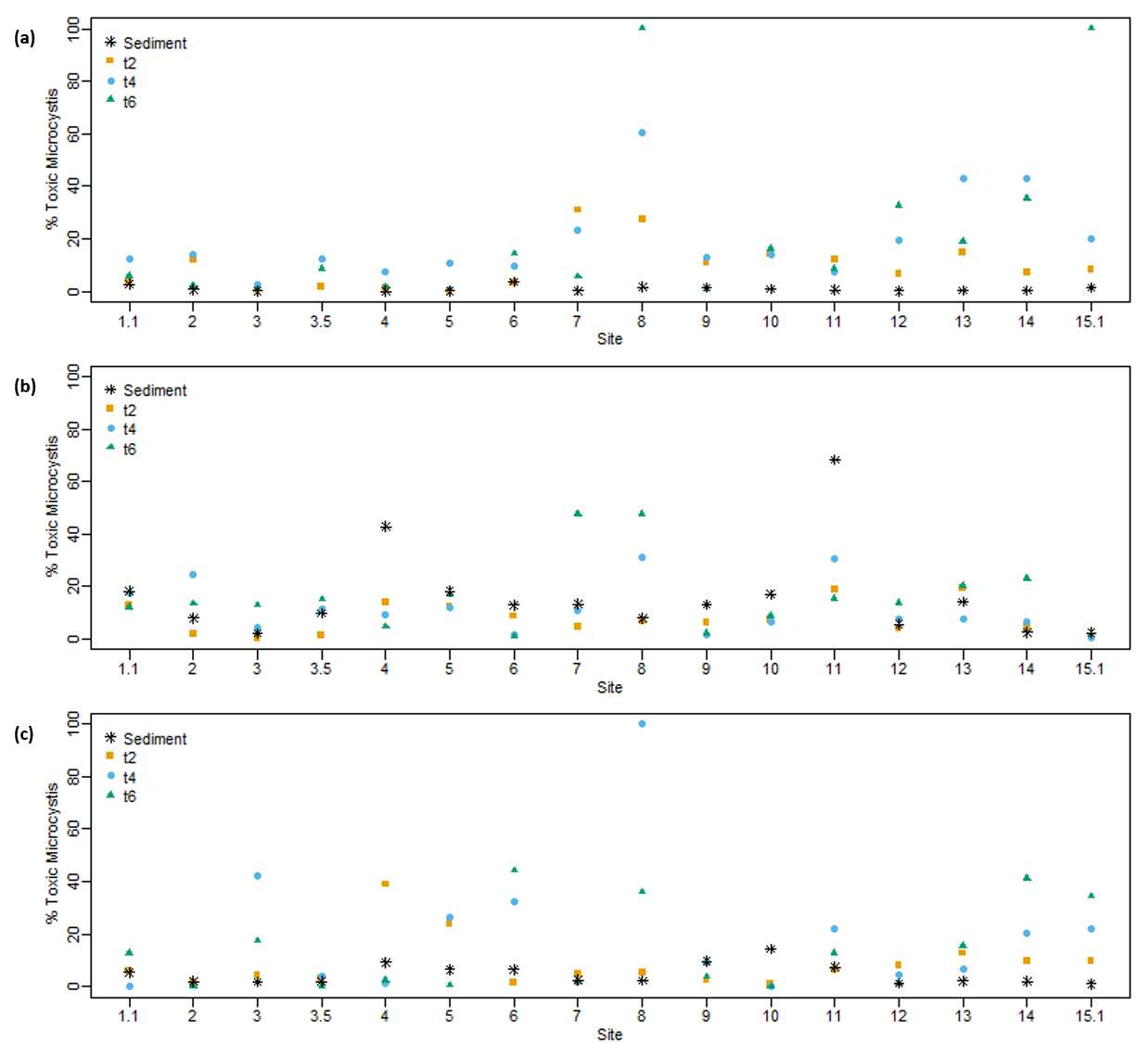

Supplement: S7 Fig — (TIF) [file pone.0206821.s007.tif]

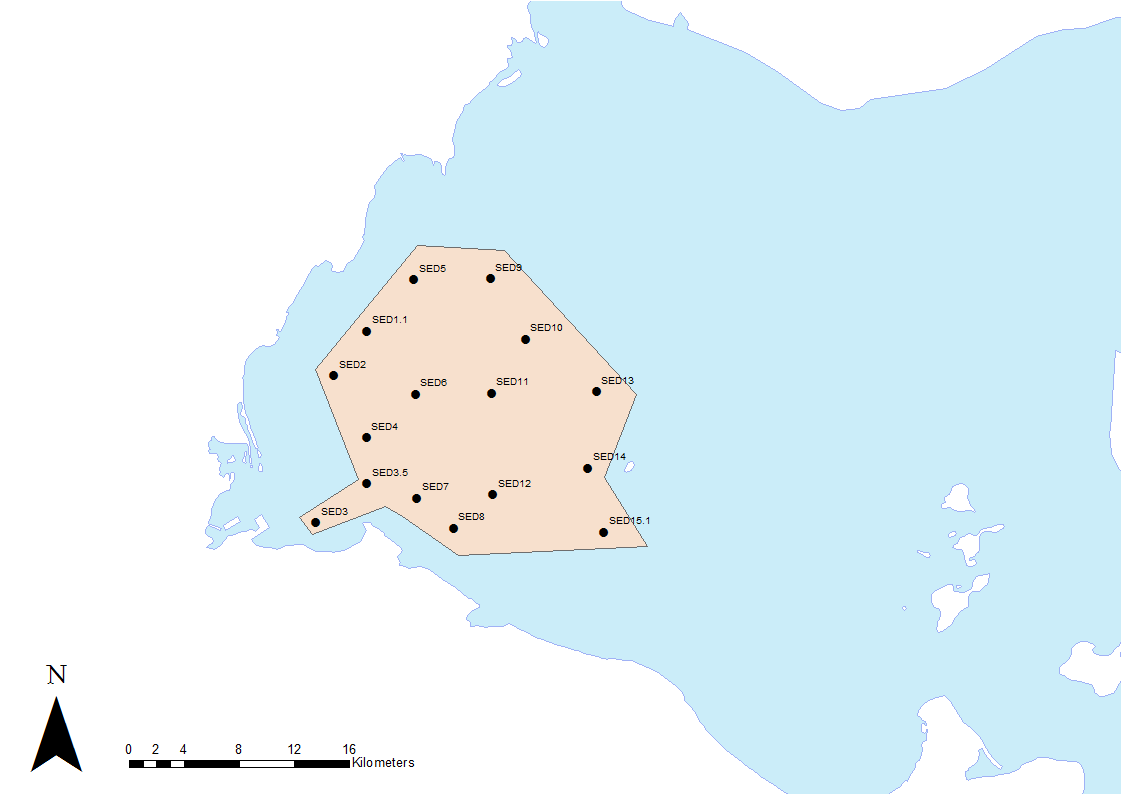

Supplement: S8 Fig — To estimate the potential contribution of Microcystis sediment recruitment to the average annual bloom, a theoretical area was established to enable quantitative comparisons between benthic and pelagic populations of Microcystis. Since values for variables and constants are developed based on the 16 sites analyzed in this study, an area encapsulating those sites was used for this analysis. The area is ~375 km2 and, assuming an average depth of 7 m, contains a water volume of 2.625 x 10^12^ L. (TIF) [file pone.0206821.s008.tif]
