# Supplementary material for: Establishing spatial and temporal patterns in Microcystis sediment seed stock viability and their relationship to subsequent bloom development in Western Lake Erie
Source: PLoS One. 2018 Nov 21;13(11):e0206821. doi: 10.1371/journal.pone.0206821 (PMC6248936; doi:10.1371/journal.pone.0206821)
Supplement: S1 File — (DOCX) [file pone.0206821.s009.docx]

#Reads in relevant documents and packages

> SedMC=read.csv(file="RDataSummary.csv", na.strings="", stringsAsFactors = FALSE)

> SedCulture=read.csv(file="RDataCulture.csv", na.strings= "", stringsAsFactors = FALSE)

> SedCombined=read.csv(file="Sed_Combined_Name.csv", na.strings= "", stringsAsFactors = FALSE)

> CultureCombined=read.csv(file="Culture_Combined_Name.csv", na.strings= "", stringsAsFactors = FALSE)

> CultureAccu=read.csv(file="RDataCultureAccumulation.csv", stringsAsFactors = FALSE)

> PerToxicMC=read.csv(file="PerToxMC.csv", stringsAsFactors = TRUE)

> PerToxicMCwSed=read.csv(file="PerToxMCwSed.csv", stringsAsFactors = TRUE)

> SedCombinedCHLA=read.csv(file="Sed_Combined_Name_Outlier_Removed.csv", na.strings= "", stringsAsFactors = FALSE)

> cbPalette <- c("#999999", "#E69F00", "#56B4E9", "#009E73", "#F0E442", "#0072B2", "#D55E00", "#CC79A7")

> cbbPalette <- c("#000000", "#E69F00", "#56B4E9", "#009E73", "#F0E442", "#0072B2", "#D55E00", "#CC79A7")

> library(car)

> library(ggplot2)

> library(lattice)

> library(ggpubr)

> library(vegan)

> library(MASS)

> library(RVAideMemoire)

#Converts data type from numeric to factor

> SedMC$Site<- as.factor(SedMC$Site)

> SedMC$Year<- as.factor(SedMC$Year)

> SedMC$Month<- as.factor(SedMC$Month)

> SedMC$GrabType<- as.factor(SedMC$GrabType)

> SedCulture$Site<- as.factor(SedCulture$Site)

> SedCulture$Year<- as.factor(SedCulture$Year)

> SedCulture$Month<- as.factor(SedCulture$Month)

> SedCulture$Time<- as.factor(SedCulture$Time)

> SedCombined$Site<- as.factor(SedCombined$Site)

> SedCombined$Event<- as.factor(SedCombined$Event)

> SedCombined2$Site<- as.factor(SedCombined2$Site)

> SedCombined2$Event<- as.factor(SedCombined2$Event)

> SedCombinedCHLA$Site<- as.factor(SedCombinedCHLA$Site)

> SedCombinedCHLA$Event<- as.factor(SedCombinedCHLA$Event)

> CultureCombined$Site<- as.factor(CultureCombined$Site)

> CultureCombined$Event<- as.factor(CultureCombined$Event)

> CultureCombined3$Time<- as.factor(CultureCombined3$Time)

> CultureAccu$Event<- as.factor(CultureAccu$Event)

> CultureAccu$Site<- as.factor(CultureAccu$Site)

> CultureAccu$Period<- as.factor(CultureAccu$Period)

> PerToxicMC$Site<- as.factor(PerToxicMC$Site)

> PerToxicMCwSed$Site<- as.factor(PerToxicMCwSed$Site)

#Log transform 16S and mcyD columns and change column names

> SedMC2 <- SedMC

> SedMC2[,8:9]<- log(SedMC2[8:9])

> colnames(SedMC2)[8:9]= c("log16S", "logmcyD")

> SedCulture[,5:6]<- log(SedCulture[5:6])

> colnames(SedCulture)[5:6]= c("log16S", "logmcyD")

> SedCombined2 <- SedCombined

> SedCombined2[,7:8]<- log(SedCombined2[7:8])

> colnames(SedCombined2)[7:8]= c("log16S", "logmcyD")

> SedCombinedCHLA2<- SedCombinedCHLA

> SedCombinedCHLA2[,7:8]<- log(SedCombinedCHLA2[7:8])

> colnames(SedCombinedCHLA2)[7:8]= c("log16S", "logmcyD")

> SedCultureApril15<- subset(SedCulture, Month=="April" & Year==2015)

> SedCultureNov15<- subset(SedCulture, Month=="November" & Year==2015)

> SedCultureApril16<- subset(SedCulture, Month=="April" & Year==2016)

> SedCultureT2<- subset(SedCulture, Time=="T2")

> CultureCombinedT2<- subset(CultureCombined, Time=="T2")

#Create a dataframe with only continuous variables

> SedMC3<- SedMC2[-1:-4]

> SedMC4<- SedMC[-1:-4]

> SedCombinedmcyD<-SedCombined3[-4]

> SedCombined16S<-SedCombined3[-5]

> CultureAccu16S<-CultureAccu[-1:-3]

> CultureAccu16S<-CultureAccu16S[-2:-4]

> CultureAccumcyD<-CultureAccu[-1:-4]

> CultureAccumcyD<-CultureAccumcyD[-2:-3]

> CultureCombined3<-CultureCombined[-4:-7]

> PerToxicMCwSed<-PerToxicMCwSed[-5:-6]

> SedMC16S<- SedMC4[-5]

> SedMCmcyD<- SedMC4[-4]

#’Compute correlations of columns of a dataframe of mixed types. Function #’created by Srikanth KS (talegari)

> cor2 = function(df){

stopifnot(inherits(df, "data.frame"))

stopifnot(sapply(df, class) %in% c("integer"

, "numeric"

, "factor"

, "character"))

cor_fun <- function(pos_1, pos_2){

# both are numeric

if(class(df[[pos_1]]) %in% c("integer", "numeric") &&

class(df[[pos_2]]) %in% c("integer", "numeric")){

r <- stats::cor(df[[pos_1]]

, df[[pos_2]]

, use = "complete.obs"

, method = "kendall"

)

}

# one is numeric and other is a factor/character

if(class(df[[pos_1]]) %in% c("integer", "numeric") &&

class(df[[pos_2]]) %in% c("factor", "character")){

r <- sqrt(

summary(

stats::lm(df[[pos_1]] ~ as.factor(df[[pos_2]])))[["r.squared"]])

}

if(class(df[[pos_2]]) %in% c("integer", "numeric") &&

class(df[[pos_1]]) %in% c("factor", "character")){

r <- sqrt(

summary(

stats::lm(df[[pos_2]] ~ as.factor(df[[pos_1]])))[["r.squared"]])

}

# both are factor/character

if(class(df[[pos_1]]) %in% c("factor", "character") &&

class(df[[pos_2]]) %in% c("factor", "character")){

r <- lsr::cramersV(df[[pos_1]], df[[pos_2]], simulate.p.value = TRUE)

}

return(r)

}

cor_fun <- Vectorize(cor_fun)

# now compute corr matrix

corrmat <- outer(1:ncol(df)

, 1:ncol(df)

, function(x, y) cor_fun(x, y)

)

rownames(corrmat) <- colnames(df)

colnames(corrmat) <- colnames(df)

return(corrmat)

}

> SedCorLog2<-cor2(SedMC3)

#'Plots correlation matrices to correlogram with a certain color scheme.

> corrplot::corrplot(SedCorLog2, method="number", type = "lower", tl.col = "black", col = c("#E69F00","#009E73"))

#'Generates distance matrix using non-Euclidean Bray Curtis method. Before #’generating matrix, I

#’omitted all rows with "NA"s in them.

> dSedMC16S <- vegdist(SedMC16S, method="bray", na.rm = TRUE)

> fitSedCombinedISO <- isoMDS(dSedMC16S, k=2)

> head(fitSedCombinedISO)

> x2 <- fitSedCombinedISO$points[,1]

> y2 <- fitSedCombinedISO$points[,2]

> plot(x2, y2, xlab="Coordinate 1", ylab="Coordinate 2", type= "n")

> text(x2, y2, labels = SedCombined2$Site,

col= c("#D55E00", "#000000", "#009E73", "#0072B2")[as.factor(SedCombined2$Event)],

cex=0.7,

font = 2)

> legend("bottomright",

border = "black",

bty= "o",

legend= c("November 2014", "April 2015","November 2015","April 2016"),

text.col = c("#D55E00", "#000000", "#009E73", "#0072B2"),

cex=0.6)

> dSedMCmcyD <- vegdist(SedMCmcyD, method="bray", na.rm = TRUE)

> fitSedCombinedISO1 <- isoMDS(dSedMCmcyD, k=2)

> head(fitSedCombinedISO1)

> x1 <- fitSedCombinedISO1$points[,1]

> y1 <- fitSedCombinedISO1$points[,2]

> plot(x1, y1, xlab="Coordinate 1", ylab="Coordinate 2", type= "n")

> text(x1, y1, labels = SedCombined2$Site,

col= c("#D55E00", "#000000", "#009E73", "#0072B2")[as.factor(SedCombined2$Event)],

cex=0.7,

font = 2)

> legend("bottomright",

border = "black",

bty= "o",

legend= c("November 2014", "April 2015","November 2015","April 2016"),

text.col = c("#D55E00", "#000000", "#009E73", "#0072B2"),

cex=0.6)

| > dSedCombinedmcyD <- vegdist(SedCombinedmcyD, method="bray", na.rm = TRUE)  > dSedCombined16S <- vegdist(SedCombined16S, method="bray", na.rm = TRUE)  > adonis2(dSedCombined16S ~ SedCombined$Site + SedCombined$Event)  Permutation test for adonis under reduced model  Terms added sequentially (first to last)  Permutation: free  Number of permutations: 999  adonis2(formula = dSedCombined16S ~ SedCombined$Site + SedCombined$Event)  Df SumOfSqs R2 F Pr(>F)  SedCombined$Site 15 3.5580 0.25791 1.1489 0.281  SedCombined$Event 3 2.3920 0.17340 3.8622 0.001 ***  Residual 38 7.8451 0.56869  Total 56 13.7951 1.00000  ---  Signif. codes: 0 ‘***’ 0.001 ‘**’ 0.01 ‘*’ 0.05 ‘.’ 0.1 ‘ ’ 1  > adonis2(dSedCombinedmcyD ~ SedCombined$Site + SedCombined$Event)  Permutation test for adonis under reduced model  Terms added sequentially (first to last)  Permutation: free  Number of permutations: 999  adonis2(formula = dSedCombinedmcyD ~ SedCombined$Site + SedCombined$Event)  Df SumOfSqs R2 F Pr(>F)  SedCombined$Site 15 2.5052 0.18546 0.8150 0.790  SedCombined$Event 3 3.2159 0.23806 5.2308 0.001 ***  Residual 38 7.7874 0.57648  Total 56 13.5085 1.00000  ---  Signif. codes: 0 ‘***’ 0.001 ‘**’ 0.01 ‘*’ 0.05 ‘.’ 0.1 ‘ ’ 1  > pairwise.perm.manova(dSedCombined16S, SedCombined$Event, p.method = "BH")  Pairwise comparisons using permutation MANOVAs on a distance matrix  data: dSedCombined16S by SedCombined$Event  999 permutations  14-Nov 15-Apr 15-Nov  15-Apr 0.026 - -  15-Nov 0.014 0.646 -  16-Apr 0.006 0.014 0.052  P value adjustment method: BH  > pairwise.perm.manova(dSedCombinedmcyD, SedCombined$Event, p.method = "BH")  Pairwise comparisons using permutation MANOVAs on a distance matrix  data: dSedCombinedmcyD by SedCombined$Event  999 permutations  14-Nov 15-Apr 15-Nov  15-Apr 0.008 - -  15-Nov 0.552 0.003 -  16-Apr 0.018 0.395 0.003  P value adjustment method: BH  > dCultureAccumcyD <- vegdist(CultureAccumcyD, method="bray", na.rm = TRUE)  > dCultureAccu16S <- vegdist(CultureAccu16S, method="bray", na.rm = TRUE)  > dPerToxicMCwSed <- vegdist(PerToxicMCwSed[-1:-3], method="bray", na.rm = TRUE)  > adonis2(dCultureAccu16S ~ CultureAccu$Event + CultureAccu$Site + CultureAccu$Period)  Permutation test for adonis under reduced model  Terms added sequentially (first to last)  Permutation: free  Number of permutations: 999  adonis2(formula = dCultureAccu16S ~ CultureAccu$Event + CultureAccu$Site + CultureAccu$Period)  Df SumOfSqs R2 F Pr(>F)  CultureAccu$Event 2 27906 0.85913 46.9555 0.211  CultureAccu$Site 15 31317 0.96415 7.0261 0.406  CultureAccu$Period 2 -63589 -1.95767 -106.9962 0.792  Residual 124 36847 1.13439  Total 143 32482 1.00000  > adonis2(dCultureAccumcyD ~ CultureAccu$Event + CultureAccu$Site + CultureAccu$Period)  Permutation test for adonis under reduced model  Terms added sequentially (first to last)  Permutation: free  Number of permutations: 999  adonis2(formula = dCultureAccumcyD ~ CultureAccu$Event + CultureAccu$Site + CultureAccu$Period)  Df SumOfSqs R2 F Pr(>F)  CultureAccu$Event 2 -70740 -1.56556 -154.026 0.965  CultureAccu$Site 15 45060 0.99724 13.082 0.065 .  CultureAccu$Period 2 42389 0.93813 92.297 0.034 *  Residual 124 28475 0.63019  Total 143 45185 1.00000  ---  Signif. codes: 0 ‘***’ 0.001 ‘**’ 0.01 ‘*’ 0.05 ‘.’ 0.1 ‘ ’ 1  > adonis2(dPerToxicMCwSed ~ PerToxicMCwSed$Event + PerToxicMCwSed$Site + PerToxicMCwSed$Time)  Permutation test for adonis under reduced model  Terms added sequentially (first to last)  Permutation: free  Number of permutations: 999  adonis2(formula = dPerToxicMCwSed ~ PerToxicMCwSed$Event + PerToxicMCwSed$Site + PerToxicMCwSed$Time)  Df SumOfSqs R2 F Pr(>F)  PerToxicMCwSed$Event 2 1.278 0.03392 3.5474 0.005 **  PerToxicMCwSed$Site 15 3.583 0.09513 1.3265 0.087 .  PerToxicMCwSed$Time 3 2.550 0.06769 4.7194 0.001 ***  Residual 168 30.256 0.80325  Total 188 37.667 1.00000  ---  Signif. codes: 0 ‘***’ 0.001 ‘**’ 0.01 ‘*’ 0.05 ‘.’ 0.1 ‘ ’ 1  > pairwise.perm.manova(dCultureAccumcyD, CultureAccu$Period, p.method = "BH")  Pairwise comparisons using permutation MANOVAs on a distance matrix  data: dCultureAccumcyD by CultureAccu$Period  999 permutations  T0T2 T2T4  T2T4 0.11 -  T4T6 0.60 0.60  P value adjustment method: BH  > pairwise.perm.manova(dPerToxicMCwSed, PerToxicMCwSed$Event, p.method = "BH")  Pairwise comparisons using permutation MANOVAs on a distance matrix  data: dPerToxicMCwSed by PerToxicMCwSed$Event  999 permutations  15-Apr 15-Nov  15-Nov 0.015 -  16-Apr 0.386 0.016  P value adjustment method: BH  > pairwise.perm.manova(dPerToxicMCwSed, PerToxicMCwSed$Time, p.method = "BH")  Pairwise comparisons using permutation MANOVAs on a distance matrix  data: dPerToxicMCwSed by PerToxicMCwSed$Time  999 permutations  Sed T2 T4  T2 0.054 - -  T4 0.006 0.036 -  T6 0.006 0.036 0.321  P value adjustment method: BH  #'Simple linear models based on Microcystis vitality and abundance based on all  #’ data as well as by seasonal subsets |
| --- |
| > lmApril2015<-lm(log(CultureCombinedT2$Culture16S) ~ log(CultureCombinedT2$Sed16S),  + data= CultureCombinedT2, subset=(Event=="15-Apr"))  > lmNov2015<-lm(log(CultureCombinedT2$Culture16S) ~ log(CultureCombinedT2$Sed16S),  + data= CultureCombinedT2, subset=(Event=="15-Nov"))  > lmApril2016<-lm(log(CultureCombinedT2$Culture16S) ~ log(CultureCombinedT2$Sed16S),  + data= CultureCombinedT2, subset=(Event=="16-Apr"))  > lmOverall<-lm(log(CultureCombinedT2$Culture16S) ~ log(CultureCombinedT2$Sed16S))  > summary(lmApril2015)  Call:  lm(formula = log(CultureCombinedT2$Culture16S) ~ log(CultureCombinedT2$Sed16S),  data = CultureCombinedT2, subset = (Event == "15-Apr"))  Residuals:  Min 1Q Median 3Q Max  -1.6071 -0.7069 -0.2601 0.7300 1.9072  Coefficients:  Estimate Std. Error t value Pr(>\|t\|)  (Intercept) 10.052509 2.903790 3.462 0.00421 **  log(CultureCombinedT2$Sed16S) 0.003912 0.185234 0.021 0.98347  ---  Signif. codes: 0 ‘***’ 0.001 ‘**’ 0.01 ‘*’ 0.05 ‘.’ 0.1 ‘ ’ 1  Residual standard error: 1.065 on 13 degrees of freedom  (1 observation deleted due to missingness)  Multiple R-squared: 3.431e-05, Adjusted R-squared: -0.07689  F-statistic: 0.000446 on 1 and 13 DF, p-value: 0.9835  > summary(lmNov2015)  Call:  lm(formula = log(CultureCombinedT2$Culture16S) ~ log(CultureCombinedT2$Sed16S),  data = CultureCombinedT2, subset = (Event == "15-Nov"))  Residuals:  Min 1Q Median 3Q Max  -1.92849 -0.93064 0.03457 0.76045 1.97052  Coefficients:  Estimate Std. Error t value Pr(>\|t\|)  (Intercept) -1.1325 3.5274 -0.321 0.7529  log(CultureCombinedT2$Sed16S) 0.6789 0.2329 2.915 0.0113 *  ---  Signif. codes: 0 ‘***’ 0.001 ‘**’ 0.01 ‘*’ 0.05 ‘.’ 0.1 ‘ ’ 1  Residual standard error: 1.211 on 14 degrees of freedom  Multiple R-squared: 0.3777, Adjusted R-squared: 0.3332  F-statistic: 8.497 on 1 and 14 DF, p-value: 0.0113  > summary(lmApril2016)  Call:  lm(formula = log(CultureCombinedT2$Culture16S) ~ log(CultureCombinedT2$Sed16S),  data = CultureCombinedT2, subset = (Event == "16-Apr"))  Residuals:  Min 1Q Median 3Q Max  -3.8337 -0.8045 -0.0654 1.3233 2.4776  Coefficients:  Estimate Std. Error t value Pr(>\|t\|)  (Intercept) 4.8167 4.3212 1.115 0.284  log(CultureCombinedT2$Sed16S) 0.1872 0.3149 0.594 0.562  Residual standard error: 1.769 on 14 degrees of freedom  Multiple R-squared: 0.02462, Adjusted R-squared: -0.04505  F-statistic: 0.3534 on 1 and 14 DF, p-value: 0.5617  > summary(lmOverall)  Call:  lm(formula = log(CultureCombinedT2$Culture16S) ~ log(CultureCombinedT2$Sed16S))  Residuals:  Min 1Q Median 3Q Max  -4.6135 -0.8507 0.2177 0.8127 3.2912  Coefficients:  Estimate Std. Error t value Pr(>\|t\|)  (Intercept) 0.7253 2.1371 0.339 0.735908  log(CultureCombinedT2$Sed16S) 0.5495 0.1439 3.820 0.000407 ***  ---  Signif. codes: 0 ‘***’ 0.001 ‘**’ 0.01 ‘*’ 0.05 ‘.’ 0.1 ‘ ’ 1  Residual standard error: 1.6 on 45 degrees of freedom  (1 observation deleted due to missingness)  Multiple R-squared: 0.2448, Adjusted R-squared: 0.228  F-statistic: 14.59 on 1 and 45 DF, p-value: 0.000407 |
|  |
| \| > \| \| --- \| |
