# Supplementary material for: Establishing spatial and temporal patterns in Microcystis sediment seed stock viability and their relationship to subsequent bloom development in Western Lake Erie
Source: PLoS One. 2018 Nov 21;13(11):e0206821. doi: 10.1371/journal.pone.0206821 (PMC6248936; doi:10.1371/journal.pone.0206821)
Supplement: S2 File — (DOCX) [file pone.0206821.s010.docx]

Based on annual western basin cyanobacteria bloom measurements summarized in Obenour et al. [1], the average annual observed bloom in Western Lake Erie from 2002-2013 is about 20000 MT of cyanobacteria. We can scale down the average annual observed bloom for our area to be 4000 MT of cyanobacteria since our area is ~1/5 the area of the entire Western Basin. Using the conversion factor between 1 CI (cyanobacteria index), cell quantity, and MT of cyanobacteria dry weight [1-2], we can determine the expected concentration of *Microcystis* cells in our area:

$$79 MT Chla*\frac{{10}^{6}g Chla}{1 MT Chla}*\frac{1 g cell dry wt}{0.006125 g Chla}*\frac{MT cell dry wt}{{10}^{6}g cell dry wt}*\frac{1 CI}{4800 MT cell dry wt}*\frac{{10}^{20}}{1 CI}*\frac{1}{2.625 x {10}^{12} L}*\frac{1 L}{{10}^{3}mL}=\frac{1.02*{10}^{5} cells}{mL}$$

To determine the mass of sediment in the top 2 cm of that same area, an approximate bulk density of sediment (~900 kg m^-3^) was generated based on measured characteristics of collected sediment samples.

If all the *Microcystis* cells in the top 0.5 cm of our area migrated into the water column, it would result in an initial average cell concentration of 1.83 x 10^5^ cells mL^-1^.

Calculated growth rates were used to predict changes in *Microcystis* concentrations given an elapsed number of days using the following formula:

$$x_{t}=x_{0}e^{\mu t}$$

, where *x*_0_ is the initial concentration of cells in the water column, *x*_t_ is the concentration of cell equivalents at time t (days), and µ is the growth rate of cells (days^-1^).

**References**

1. Obenour DR, Gronewold AD, Stow CA, Scavia D. Using Bayesian hierarchical model to improve Lake Erie cyanobacteria bloom forecasts. Water Resour. Res. 2014; 50: 7847-7860.

2. Stumpf RP, Wynne TT, Baker DB, Fahnenstiel GL. Interannual Variability of Cyanobacterial Blooms in Lake Erie. PLoS ONE. 2012; 7(8).
